# Impedance Analysis of Thin Films of Organic-Inorganic Perovskites CH_3_NH_3_PbI_3_ with Control of Microstructure

**DOI:** 10.1186/s11671-018-2509-2

**Published:** 2018-04-12

**Authors:** Oleg V’yunov, Anatolii Belous, Sofiia Kobylianska, Leonid Kovalenko

**Affiliations:** Vernadsky Institute of General and Inorganic Chemistry of the National Academy of Sciences of Ukraine, Prospect Palladina, 32/34, Kiev, 03142 Ukraine

**Keywords:** Metal halide perovskite, Film, Microstructure, Complex impedance, I-V curve, 81.07.Pr, 81.07.–b, 84.37.+q, 72.40.+w

## Abstract

The effect of starting reagents (PbI2:{CH_3_NH_3_I + CH_3_NH_3_Cl}) with different ratios in raw solutions on the microstructure of films of organic-inorganic perovskites CH_3_NH_3_PbI_3-x_Cl_x_, as well as on the electrical properties, has been investigated. It was found that the crystallinity is increased sharply when the ratio of the starting reagents increases from 1:1 to 1:2 and is changed slightly with a further increase of ratio to 1:3. It is shown that when the ratio of starting reagents varies, the morphology of the films changes; at a ratio of 1:1, the films consist of needle-like particles, and when the ratio is increased, particles become roundish and then faceted. Additionally, the average grain size is decreased. Complex impedance curves and I-V curves have been investigated for samples with different ratios of the starting reagents. With increasing this ratio, the concentration of charge carriers remains unchanged, the mobility of charge carriers decreases, and conductivity passes through a maximum at a ratio of 1:2. The electrical properties of film are the highest at the ratio of starting reagents 1:2 due to the effect of two competing factors: the growth of crystallinity and the decrease of grain size.

## Background

The interest to hybrid organic-inorganic halides with perovskite structure has been increasing in recent years, which is due to successful attempts to increase the power conversion efficiency (PCE) into electrical energy in solar cells [1]. At the present time, in the halide system APbX_3_ (A = CH_3_NH_3_, X = Cl, Br, I), a PCE of over 22% has been attained [2], which is higher of commercial monocrystalline silicon solar cells. The relatively easy [3] and low-cost production [4] of organic-inorganic hybrid perovskites should also be pointed out, which makes them promising for the creation of highly efficient and cheap solar cells. A considerable attention in the creation of solar cells is given to the problems of ultraviolet degradation and thermal decomposition [5]. The first problem is related to charge recombination at the interface between the electrode and perovskite, where structural defects act as recombination centers [6]. The formation of a large number of defects is caused by their low formation energy [7]. Simultaneous surface passivation of the perovskite/electrode interface and increasing the stability of the perovskite structure could increase the performance of solar cells. The structure imperfection can be reduced by the partial substitution of iodide ions with chlorine ions [8] or bromine ions [9]. At the same time, it was found that the grain boundaries do not enhance charge carrier recombination and can even facilitate charge separation processes [10, 11]. The ratio of the contributions of the grain interior and grain boundaries changes with grain size [12, 13]. Considerable changes in the microstructure of films are observed on changing stoichiometric ratio CH_3_NH_3_I:PbI_2_ in initial solutions, which are used for the synthesis of organic-inorganic CH_3_NH_3_PbI_3_ halides [14, 15]. The investigation of electrical characteristics (e.g., impedance spectroscopy) of the grain interior and grain boundaries of hybrid perovskites in solar cells is complicated because of the hysteresis effect [16]. This phenomenon is attributed to the accumulation of charge carriers at the interface between contacts. In this case, an inductive loop and negative capacitance at medium and low frequencies are being observed [17]. To reduce the influence of this effect, measurements can be made using planar electrodes. However, there are no data on the electrical characteristics of the grain interior and grain boundaries of perovskites (which differ significantly in the microstructure) determined by the complex impedance method using planar electrodes.

In this work, the effect of starting reagents ({CH_3_NH_3_I + CH_3_NH_3_Cl}:PbI_2_) with different ratios in raw solutions on the microstructure of films of organic-inorganic perovskites CH_3_NH_3_PbI_3-x_Cl_x_, as well as on the electrical properties of grains and grain boundaries, has been investigated.

## Methods

### Methods of Synthesis

Lead iodide PbI_2_, methylammonium chloride CH_3_NH_3_Cl (chemically pure), and pre-synthesized methylammonium iodide CH_3_NH_3_I [18] were used as starting reagents. Dried dimethylformamide (DMF, chemically pure) was used as the solvent.

For the deposition of CH_3_NH_3_PbI_3-x_Cl_x_ films, the starting reagents PbI_2_, CH_3_NH_3_I, and CH_3_NH_3_Cl in stoichiometric ratios were dissolved in DMF and stirred at 70 °C for 1 h. Synthesis was carried out in a dry box. The resulting solution (room temperature) was applied to glass substrates by the spin-coating method. The rotation speed of the substrate was 40 rps. The thermal treatment of the films was carried out on the pre-heated hot plate in a temperature range of 70–150 °C for 30 min. The synthesis of organic-inorganic perovskites CH_3_NH_3_PbI_3-x_Cl_x_ was carried out at different ratios of the starting reagents PbI_2_ and CH_3_NH_3_I (1:1, 1:2, 1:3).

### Characterization

The phase composition was identified by X-ray powder diffractometry using a DRON-4-07 diffractometer (Cu*K*α radiation). The microstructure was studied using a microinterferometer MII-4 and a scanning electron microscope SEC miniSEM SNE 4500MB. The elemental composition of the films was studied using an EDAX Element PV6500/00 F spectrometer, which is included in the set of this microscope.

The electrical characteristics were investigated at alternating current at room temperature in the dark and with a change in the illumination up to 10 mW/cm^2^ (corresponding to 0.1 of solar illuminance on a bright day), increasing the voltage from 0 to 40 V. Xe radiation from an Infolight H3 lamp (Akodgy, Seoul, South Korea) with a power of 50 W was used. The illumination was determined using a Lux/FC Light Meter DL-204. The complex impedance *Z* = *Z*′ + i*Z*″ (where Z′ and Z″ are the real and imaginary parts of complex impedance) in a wide frequency range (1 Hz–1 MHz) was determined using a 1260A Impedance/Gain-Phase Analyzer (Solartron Analytical). The equivalent circuit and the values of its components were determined using ZView® for Windows (Scribner Associates Inc., USA).

## Results and Discussions

**Fig. 1:**
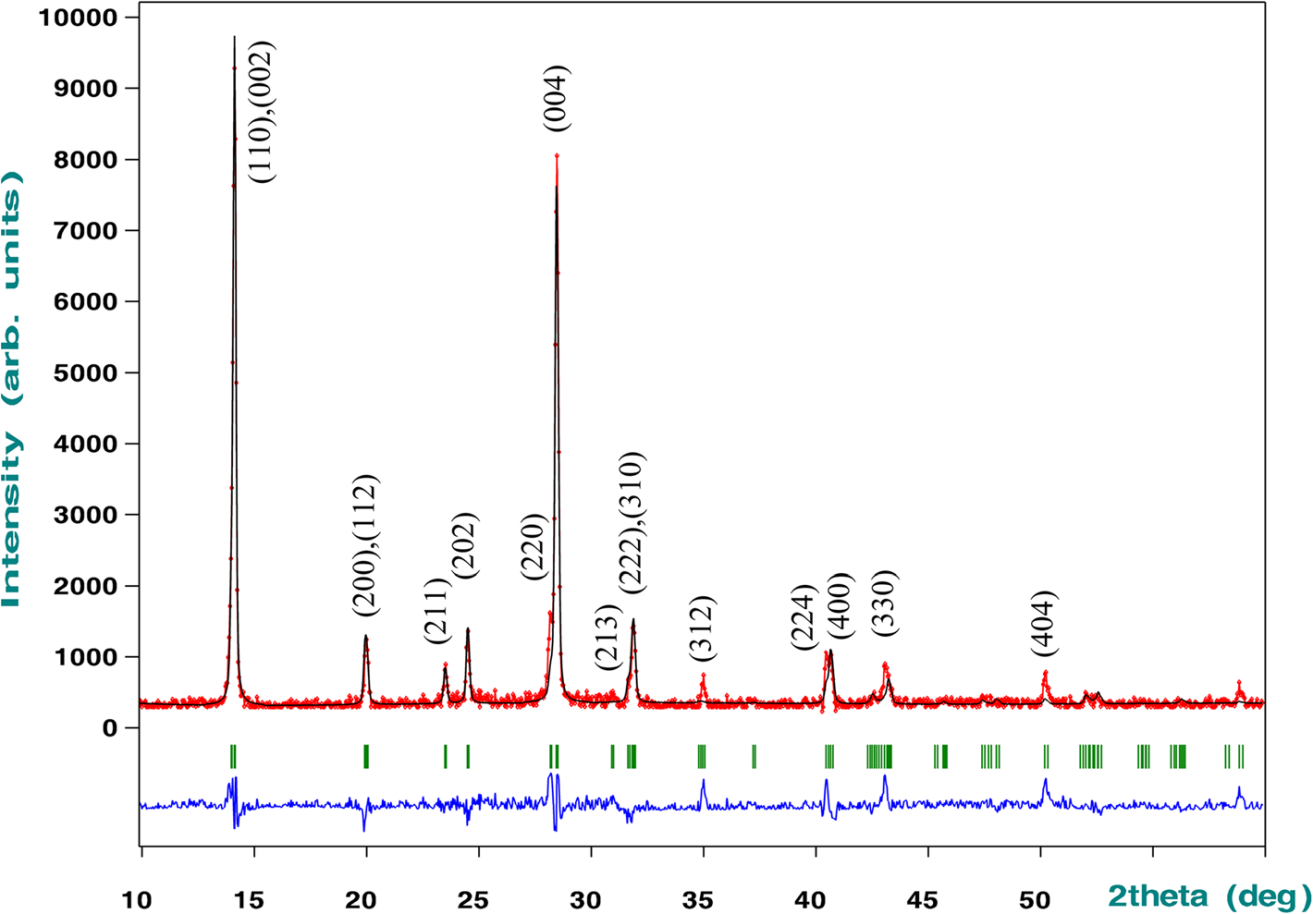
Experimental (points) and calculated (lines) X-ray powder diffraction patterns of the CH_3_NH_3_PbI_2.98_Cl_0.02_ films after heat treatment at 80 °С and the ratio of starting reagents (PbI_2_ and CH_3_NH_3_І) 1:2. Vertical bands indicate the positions of the peaks; the Miller indices are in parentheses. The difference curve is shown below

Organic-inorganic perovskites CH_3_NH_3_PbI_2.98_Cl_0.02_ were synthesized at different ratios of the starting reagents PbI_2_ and CH_3_NH_3_I: PbI_2_ + 0.98CH_3_NH_3_I + 0.02CH_3_NH_3_Cl (referred to as 1:1), PbI_2_ + 1.98CH_3_NH_3_I + 0.02CH_3_NH_3_Cl (1:2), and PbI_2_ + 2.98CH_3_NH_3_I + 0.02CH_3_NH_3_Cl (1:3); methylammonium iodide was partially substituted by 2, 1, and 0.67 mol% of CH_3_NH_3_Cl. At the ratio 1:1, the sample is single-phase after heat treatment at 80 °C but contains the PbI_2_ phase at 150 °C, which is due to the decomposition of organic-inorganic perovskite. At the ratio 1:3, the sample contains remnants of additional phase at 80 °C, which are removed by heat treatment at 150 °C. At a ratio of 1:2, the sample is single-phase in a wide temperature range. The X-ray pattern of the sample corresponds to tetragonal symmetry (space group I4/mcm, No. 140) with the coordinates of atoms: Pb (4c) 0 0 0, I1 (8h) x y 0, I2 (4a) 0 0 ¼, C (16l) x y z, and N (16l) x y z [19]. Using the Rietveld full-profile analysis (Fig. 1), the unit cell parameters were refined (*a* = 0.8870(2) nm, *c* = 1.2669(8) nm, *V* = 0.9968(7) nm^3^), which agrees with literature data [19].

**Fig. 2:**
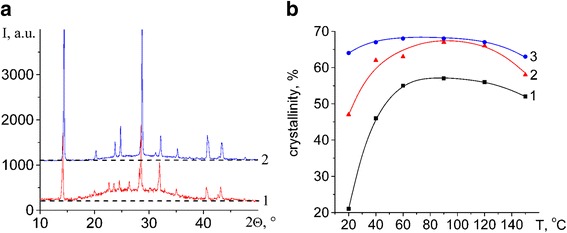
**a** Comparison of XRD patterns of organic-inorganic films CH_3_NH_3_PbI_2.98_Cl_0.02_ synthesized at ratios of PbI_2_ and CH_3_NH_3_I deposited at 20 °C (1) and 90 °C (2). **b** Film crystallinity as a function of deposition temperature of organic-inorganic films CH_3_NH_3_PbI_2.98_Cl_0.02_ synthesized at ratios of PbI_2_ and CH_3_NH_3_I 1:1 (1), 1:2 (2), and 1:3 (3) (lines are drawn in for clarity)

The percent crystallinity for each film was estimated by the ratio of the area under each crystalline peak to the total area in the XRD spectra (Fig. 2a). Plots of the percent crystallinity as a function of deposition temperature of organic-inorganic films CH_3_NH_3_PbI_2.98_Cl_0.02_ synthesized at ratios of PbI_2_ to CH_3_NH_3_I 1:1 (1), 1:2 (2), and 1:3 (3) are shown in Fig. 2b. The increasing temperature from room temperature to ~ 60 °C increases crystallinity. In the range of 60–120 °C, the crystallinity does not change significantly. A further increase in temperature decreases the crystallinity due to the disproportionation and PbI_2_ separation. In the temperature range of 60–120 °C, the crystallinity is increased sharply with the ratio of the starting reagents from 1:1 to 1:2 (Fig. 2b, curves 1 and 2) and then is changed slightly (Fig. 2b, curves 2 and 3). Therefore, the crystallinity can significantly affect the properties of the films.

**Fig. 3:**
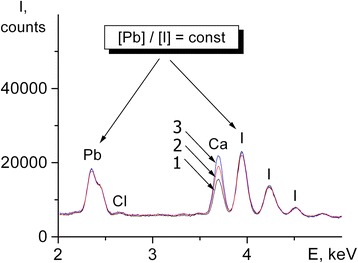
EDX of CH_3_NH_3_PbI_2.98_Cl_0.02_ films after heat treatment at 80 °С and the ratio of starting reagents (PbI_2_ and CH_3_NH_3_І) 1:1 (1), 1:2 (2), and 1:3 (3)

The elemental composition of the CH_3_NH_3_PbI_2.98_Cl_0.02_ films deposited from solutions with different ratios of the starting reagents PbI_2_ and CH_3_NH_3_I (1:1, 1:2, and 1:3) was studied by the energy-dispersive X-ray spectroscopy (EDX) method (Fig. 3). The spectrum exhibits peaks of Ca, which is contained in the glass substrate [20]. It is seen from Fig. 2 that the intensity ratio of the Pb and I peaks is the same for the samples at different ratios of PbI_2_ and CH_3_NH_3_I.

**Fig. 4:**
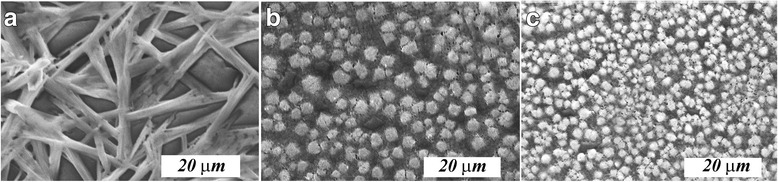
Microstructure of films CH_3_NH_3_PbI_2.98_Cl_0.02_ after heat treatment at 80 °С and the ratio of starting reagents (PbI_2_ and CH_3_NH_3_І) 1:1 (**a**), 1:2 (**b**), and 1:3 (**c**)

The shape and particle size of the obtained CH_3_NH_3_PbI_2.98_Cl_0.02_ films strongly depend largely on the stoichiometric ratio of the starting reagents. At the ratio PbI_2_:CH_3_NH_3_I = 1:1, the films consist of needle-like particles, which are arranged along the substrate plane (Fig. 4). In the case of PbI_2_:CH_3_NH_3_I = 1:2, roundish particles have been obtained (Fig. 4a). When the amount of methylammonium iodide is further increased (PbI_2_:CH_3_NH_3_I = 1:3), a conversion from roundish particles to faceted particles is observed (Fig. 4b). In this case, the film thicknesses at different ratios of the starting reagents and at a heat treatment temperature of 80 °C are close together (900 nm).

**Fig. 5:**
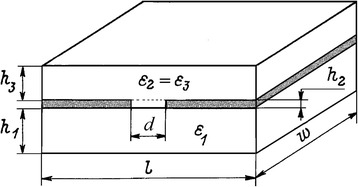
The scheme of the measured multilayer system consisting of a substrate (*l* = 16 mm, *w* = 24 mm, *h*_1_ = 1 mm), on which electrodes of thickness *h*_2_ = 90 nm were deposited at a distance *d* = 250 μm, and the film under investigation was *h*_3_ = 500 nm thick

**Fig. 6:**
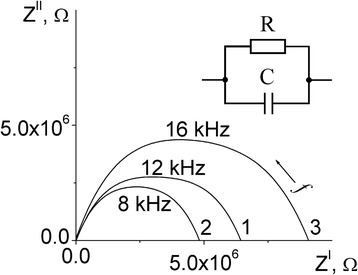
Complex impedance diagrams (Nyquist plots) and equivalent circuit of multilayer system consists of perovskite organic-inorganic films prepared at the ratio of starting reagents (PbI_2_ and CH_3_NH_3_І) 1:1 (1), 1:2 (2), and 1:3 (3) on glass substrate at an illumination of 30 klx. Measurements performed at a voltage of 1 V in a dry atmosphere. The numbers above the curves are the frequency (Hz)

The complex impedance data were collected on the day of synthesis, since the microstructure and properties of the samples may change during storage [21]. In the air atmosphere, a contribution of ionic conductivity appears, which manifests itself in the complex impedance spectra as an additional inclined line, which is characteristic of blocking electrodes [22, 23]. In order to avoid moisture and additional ionic conductivity, the measurements were made in a dry (humidity ≤ 7 ppm) nitrogen atmosphere [24]. For measurements, the film was deposited on a substrate with pre-applied electrodes (Fig. 5). The impedance curves of the multilayer system consist of organic-inorganic films deposited on glass substrate, which were measured in a dry atmosphere, are typical of materials characterized only by electronic conductivity (Fig. 6). The complex impedance diagram contains one semicircle in the medium frequency range (8 kHz–80 Hz), which can be described by an equivalent circuit consisting of a capacitor and resistor connected in parallel [25]. In the analysis, additional elements simulating the resistance of current-carrying parts and substrates were added; the parameters of which were determined by measuring the cell without deposited film.

The film parameters (dielectric constant and current density) were calculated using the partial capacitance method [26]. According to this approach, the measured multilayer system was represented as three simple planar capacitors with uniform filling and connected in parallel. For normal electric field components at the film interfaces, zero boundary conditions were assumed. The deposited film was conditionally divided into two parts (Fig. 5): the inner parallelepiped (width *d* and thickness *h*_2_) and the outer parallelepiped (width *l* and thickness *h*_3_). The capacity of the multilayer system (*C*) can be found as the sum of three partial capacitances *C* = *C*_1_ + *C*_2_ + *C*_3_, where *C*_1_, *C*_2_, and *C*_3_ are the capacitances of the parts of the planar capacitor, which are scattering fields in (1) the substrate, (2) the inner parallelepiped of the film, and (3) the outer parallelepiped of the film. The capacitance of the inner part of the film (part 2) is determined by the usual equation of a flat capacitor, ${C}_2=\frac{{\varepsilon \varepsilon}_0\left(w\times {h}_2\right)}{d}$. The capacity of the substrate (part 1), as well as the capacity of the outer part of the film (part 3), was determined using the Schwarz-Christoffel conformal mapping transformation adapted by Gevorgian [27]. According to this method, the ellipse of electric fields in the sample is conditionally transformed into a rectangle. In this case, the capacitance of the substrate will be expressed by the formulas ${C}_1=\frac{{\varepsilon \varepsilon}_0K\left({k}^{\prime}\right)}{2K(k)}$ and ${k}^{\prime }=\sqrt{1-{k}^2}$, where *K* (*k*) is a complete elliptic integral of the first kind; *k* is the modulus of the elliptic integral; *ε*_0_ is the permittivity of free space; and *ε*_r_ is the relative permittivity of the substrate. Glass of E class (radio engineering) with low dielectric loss and *ε* = 6.6 was used as the substrate [28]. To solve elliptic integrals, we used the approximation proposed in [29]. Using a similar formula, the capacity of the outer part of the film was calculated. The experimental permittivity *ε* = 52 was determined, and this value is in agreement with the published data. The calculations based on density functional theory and density functional perturbation theory showed that the optical contribution to permittivity is *ε*_∞_ = 5.6–6.5, and the dielectric contribution is *ε*_0_ = 18.0–37.3 for the low-temperature cubic phase (Pm-3 m) [30]. Direct measurements yielded *ε* ~ 15–18 for low-temperature cubic phase (Pm-3 m) and *ε* ~ 60 for the room temperature tetragonal phase (I4/mcm) [31].

**Fig. 7:**
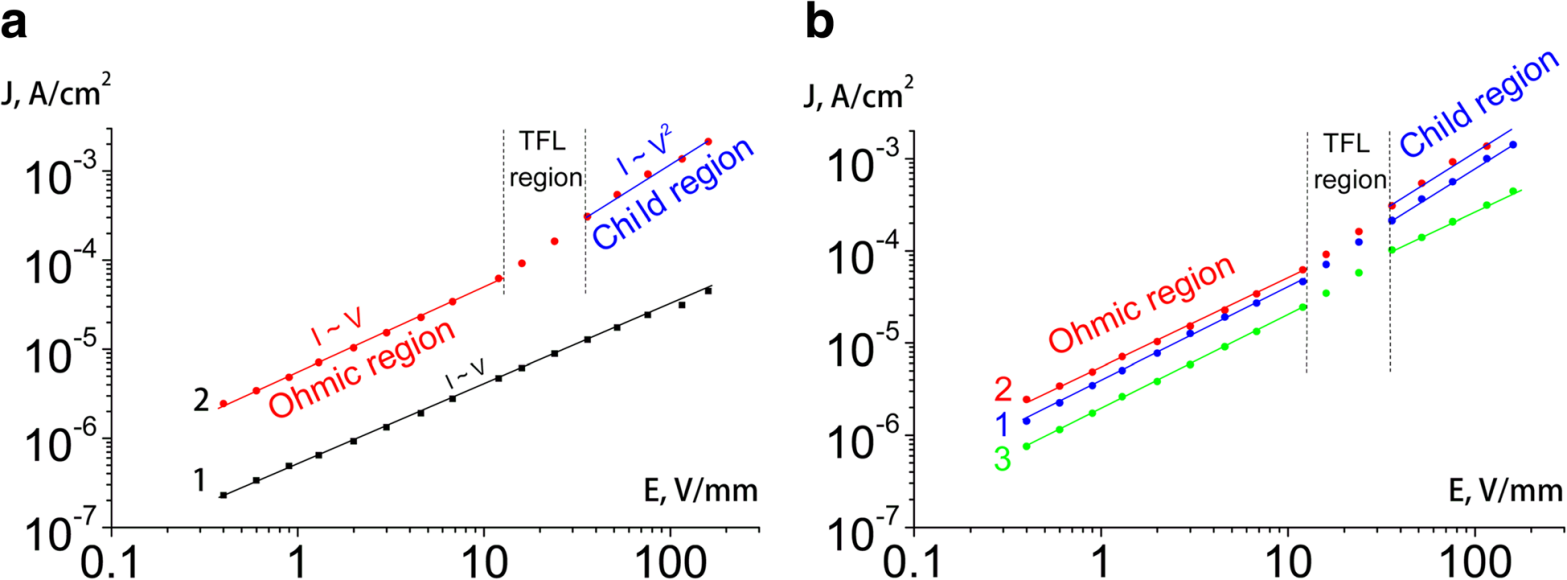
Dependence of the current density on the voltage of **a** the organic-inorganic prepared at the ratio of starting reagents (PbI_2_ and CH_3_NH_3_І) 1:2 at different illumination levels, 0 (1) and 30 klx (2), and **b** the organic-inorganic film prepared at the ratio of starting reagents 1:1 (1), 1:2 (2), and 1:3 (3) at an illumination of 30 klx

Figure 7 shows the current density calculated from impedance data vs voltage applied to organic-inorganic films. The dark current is linearly dependent on the applied voltage, while under illumination, several linear regions are observed (Fig. 7). Previously, three regions were observed on the I-V curve of a single-crystal organic-inorganic perovskite, which were described as a change from the ohmic region to the trap-filled limit (TFL) region, and further to the Child region [32]. These regions can be observed at voltages of tens of volt per millimeters (depending on the sample and the type of electrode) and can be used to calculate the characteristics of charge carriers (namely, density and mobility) [33]. In particular, the dependence of current (*I*) on electric field (*V*) in the Child region is described by the equation *j* = (9/8)*εμV*^2^/*d*^3^ (where *ε* is the permittivity of the sample, *μ* is the mobility of charge carriers, *d* is the distance between the electrodes), which makes it possible to determine the mobility of charge carriers. In the ohmic region, the current-voltage dependence is described by the equation *j* = *eμnV*/*d* (where *n* is the density of charge carriers). Using the previously calculated mobility (in the Child region) of charge carriers, the density of charge carriers can be determined.

The Child law describes the current flow limited by a space charge in the mobility mode (trap-free quadratic relation) and is observed in dielectric materials that contain no traps [34]. When a relatively low voltage is applied to an unilluminated film, the density of injected carriers is small relative to the density of traps. So current-voltage curve in the investigated range of voltages obeys the linear Ohm’s law (Fig. 7a, curve 1). Under high illumination, photo-generated carriers deactivate the trapping defects, and at a sufficiently high voltage, a trap-free mobility mode is observed, and the dependence obeys the quadratic Child law (Fig. 7, curve 2) [35].

As can be seen from Fig. 7b, the organic-inorganic film obtained with a ratio of the starting reagents of 1:2 has the maximum conductivity among the samples investigated. In addition, an increase in the ratio of the starting reagents results in a decrease in charge carrier mobility. The decrease in the slope of the plot in the Child region confirms this fact. The same slope in the ohmic region at the same level of illumination indicates a close amount of charge carriers generated.

## Conclusions

It has been shown that when the ratio of the starting reagents (PbI_2_:CH_3_NH_3_I) is changed, the crystallinity and morphology of films changes. In particular, the crystallinity is increased sharply when the ratio of the starting reagents increases from 1:1 to 1:2 and is changed slightly with a further increase of ratio to 1:3. At the ratio of the starting reactants 1:1, the films consist of needle-like particles, which are arranged along the substrate plane. When the methylammonium iodide content is increased, a conversion to roundish and then to faceted particles is observed. Additionally, the average grain size is decreased. Inclined lines on the complex impedance plots of samples measured in the air atmosphere (humidity ~ 65%) are associated with the appearance of ionic conductivity in a liquid dielectric. In the case of measurements in a dry atmosphere, three regions were observed on the I-V curve obeying Ohm’s law, the trap-filled limit, and the Child law. With an increase in the ratio of the starting reagents, the mobility of the charge carriers decreases, and the conductivity passes through a maximum at a ratio of 1:2. At the same level of illumination, the same number of charge carriers was generated. The electrical properties of the film are highest at the ratio of starting reagents 1:2 due to the effect of two competing factors: the growth of crystallinity and the decrease of grain size.

## Abbreviations

CPE: Constant phase element
DC: Direct current
DMF: Dimethylformamide, C_3_H_7_NO
EDX: Energy-dispersive X-ray spectroscopy
I-V curves: Current-voltage curve
PCE: Power conversion efficiency
SEM: Scanning electron microscopy
XRD: X-ray diffraction
